# The effect of municipality-level social media use on youth mental health

**DOI:** 10.1186/s12889-025-24727-4

**Published:** 2025-10-14

**Authors:** Olav Bertin Tveit, Guido Biele

**Affiliations:** Department of Child Health and Development, Norwegian Institute of Public Health, P.O. Box 222 Skøyen, Oslo, 0213 Norway

**Keywords:** Social media, Youth mental health, Ecologic analysis, Causal inference

## Abstract

**Background:**

Rising internalizing problems (e.g., depression and anxiety) among youth, particularly among females, have raised concerns about potential societal causes. Social media use (SMU) has emerged as a key focus, given its widespread adoption since the early 2010s. While small to moderate correlations are typically reported between SMU and internalizing problems on the individual level, a complementary community-based perspective allows for assessing the effects of living in environments with high or low social media use on youth mental health.

**Methods:**

This study investigates the effect of SMU on internalizing symptoms among Norwegian youth in a longitudinal study at the municipality level. The study uses data from the nationwide Ungdata surveys (2014–2024), covering 528 cohorts across 181 municipalities, comprising 40 014 individual responses. Anxiety and depressive symptoms were assessed using items adapted from the Hopkins Symptom Checklist and the Depressive Mood Inventory, respectively. We applied a Bayesian multilevel model, accounting for time-varying and time-constant confounders.

**Results:**

An additional hour of average SMU corresponded to a 0.70 [0.26, 1.14] SD increase in anxiety scores for boys but showed no clear association for girls (-0.03 [-0.31, 0.24]). For depressive symptoms, a one-hour increase in average SMU corresponded to an increase of 0.25 [0.03, 0.46] for boys, with no clear effect for girls (0.02 [-0.15, 0.19]). The models accounted for a substantial proportion of variance in T2 outcomes (r² = 0.6 − 0.8).

**Conclusion:**

Assuming that all relevant factors influencing both social media use and youth mental health were accounted for, the findings suggest that living in municipalities with high social media use may have a small effect on youth mental health among boys, while no effect was observed among girls.

**Supplementary Information:**

The online version contains supplementary material available at 10.1186/s12889-025-24727-4.

## Introduction

The prevalence of internalizing problems (i.e., depression and anxiety) among youths has increased in recent decades, particularly among females [1, 2]. One of the most significant societal changes since the early 2010 s has been the widespread adoption of smartphones and social media; for example, more than a third of Norwegian youth report using social media for over three hours per day [3]. Excessive social media use (SMU) is associated with several adverse mental health outcomes in youths, such as depression, anxiety, suicidal ideation, and sleep problems [4]. However, there is still considerable debate in public and academic spheres regarding social media’s role in shaping current mental health trends [5–7]. Associations and effects diverge considerably, depending on the type of analysis. Cross-sectional meta-analyses generally give a correlation in the range of *r* =.05-0.17 between social media use and depression [7]. Longitudinal studies typically show slightly smaller associations [8], and a recent meta-analysis of experimental studies concluded that there is no consistent evidence that SMU is harmful to youth [5].

Most research on the link between SMU and youth mental health has focused on individual-level associations or effects. For example, many studies have examined whether differences in outcomes can be explained by whether social media is used actively (e.g., posting messages) or passively (e.g., scrolling), although evidence for consistent differences in their association with anxiety and depression has been limited [9, 10]. While individual-level studies are essential for identifying dose-response relationships, they may fail to capture determinants of ill-being to which most of the population is exposed [11]. Widespread use of a medium in a group can affect the social fabric, also for non-users [12]. For instance, while youths as a group engage in less face-to-face socialization than in previous decades, social media use is positively correlated with in-person socializing at the individual level [13]. Because SMU is ubiquitous it is an integral part of young people’s social lives, the cost of abstaining from SMU in a group where the majority are online may thus outweigh the benefits, even if some types of SMU are detrimental to mental health [12].

Identifying all effects of social media on youth as a group requires inclusion of both individual-level and aggregate-level ecologic perspectives [11]. Such studies remain relatively rare, despite a few notable examples [14–16]. Results from these studies indicate that the mental health of young people deteriorated after the introduction of Facebook to US colleges [15] or high-speed internet in Spanish regions [14], with stronger effects for females. Aggregate-level studies face distinct methodological challenges, particularly when attempting to make inference about individual-level effects from ecologic data [17, 18]. Nonetheless, analyzing mental health trends at the regional level is valuable for public health policy, as many interventions—such as school-based restrictions or community-wide initiatives—are implemented at this scale [19, 20].

At the heart of the SMU-mental health debate lies a causal question. Identifying causal effects requires clarity about the direction of effects and assuming no unobserved confounders [21]. This makes causal inference from observational data inherently challenging and a matter of subject matter debate, as reverse causality in cross-sectional studies and control of all common causes cannot be verified empirically [21]. For example, Nilsen and colleagues [16] showed that rising SMU partially statistically explained the concurrent increase in physical health complaints among Norwegian youth. They used a large repeated cross-sectional municipality-level dataset and sophisticated multilevel models to control for time invariant confounders. However, as they investigated concurrent associations, their analysis left the directionality question unresolved. There is a need for large-scale, causally informed longitudinal studies that approach the issue with several methodologies and analytic perspectives [7, 22].

This study investigates the longitudinal effects of social media use on symptoms of depression and anxiety among Norwegian youth at the municipality level. Recognizing that unobserved confounding is an important challenge for causal inference from observational data, we chose a study design that implemented a longitudinal design and removed all time-varying unobserved confounding prior to the measurement of SMU by adjusting for the pre-treatment levels of the outcome, and we adjusted for time-constant confounders by estimating municipality level random effects.

## Methods

The data were derived from the Ungdata surveys, a nation-wide study implemented annually at the municipality level for 8th to 13th graders, when students are between 13 and 19 years of age. Participation in the Ungdata study was voluntary, and participating students gave their informed consent and could withdraw at any time. Parents were also informed and had the option to withdraw their children from the study. Participants completed an electronic questionnaire in class. The average response rate across municipalities ranged from 78 to 85% [3]. The unit of analysis was cohorts within municipalities that had participated in the survey twice, with intervals of one, two, or three years. The term ‘cohort’ is used to refer to students within a municipality who were at the same grade-level in a given year. Criteria for inclusion in the present study were that (1) Ungdata had information about the exposure SMU and the outcomes depression or anxiety at two time point, and (2) the number of participants from the cohort at the second measurement did not deviate by more than 15% from the sample size at the first measurement. This study used data from 528 cohorts from 181 municipalities, collected between 2014 and 2024. The study sample comprises a subset of the Ungdata study population, as not all municipalities implement the survey yearly, and municipalities differ in the frequency and grades for which the survey is implemented.

### Measures

The Ungdata study uses mental health items adapted from the Hopkins Symptom Checklist [23] and the Depressive Mood Inventory [24]. Depressive symptoms during the previous week were assessed with six items (“Felt that everything is a struggle”, “Had sleep problems”, “Felt unhappy, sad or depressed”, “Felt hopelessness about the future”, “Felt stiff or tense”, “Worried too much about things”). Anxiety during the previous week was assessed with three items (“Suddenly felt scared for no reason”, “Felt constant fear or anxiety”, “Been nervous or felt uneasy”). All depression and anxiety items were rated on a 4-point Likert scale (1 = *Not been affected at all*, 4 = *Been affected a great deal*). Short versions of the scales used in the present study have previously shown good psychometric properties in the Norwegian population [25]. The scales showed good internal consistency in the present sample, with Cronbach’s Alpha = 0.89 for the depression items and 0.87 for the anxiety items. Mean scores were calculated for all individuals who responded to at least 50% of the items from a scale. Municipality-cohort level data were calculated as averages over all individuals for which data was available. The sample size per cohort was calculated as the number of individuals for which exposure and outcome data was available. Individuals without either exposure or outcome data were removed from the analyses.

Social media use was assessed with the question “Think about what you do on a normal day. How much time do you spend on social media (Facebook, Instagram, etc.)?” with response options 1 = No time, 2 = Less than 30 min, 3 = 30 min–1 h, 4 = 1–2 h, 5 = 2–3 h, 6 = more than 3 h. To facilitate analysis, response categories were recoded to reflect the midpoint of each time interval in hours or minutes (e.g., 1–2 h was recoded as 1 h 30 min), with the final category (more than 3 h) recoded as 4 h.

The birth year of students was inferred from the grade-level variable, since in the Norwegian school system almost all children start school at the same age and no children repeat grades. A measure of socioeconomic status was provided by Ungdata. It was constructed as a continuous variable ranging from 0 to 3, computed using items relating to parental education, family affluence, and the number of books in the student’s home. The measure is moderately correlated with subjective wealth and students’ grades [26]. Urbanicity of municipalities was accounted for by an urbanicity index provided by Statistics Norway, based on the number of residents, places of work, availability of services, and travel times in municipalities [27]. Due to the intervals at which the Ungdata survey is implemented, the study includes relatively few cohorts from the most urban municipalities (i.e., Oslo and neighboring areas). See supplementary materials for a complete list of survey items used in the study.

From 2020 onward, anxiety items were removed from the core section of the survey, leading to a reduced number of municipalities with post-2020 anxiety data. As a result, the analytic sample included 262 cohorts across 113 municipalities for anxiety, and 528 cohorts across 181 municipalities for depression. See Table 1 for a description of the sample.

**Table 1:** Sample sizes of municipalities and cohorts

Years from T1 to T2	School grade T1	*N* cohorts		*N* unique municipalities		*N* students	
		Anxiety	Depression	Anxiety	Depression	Anxiety	Depression
1	8	11	38	6	24	419	1502
1	9	17	43	10	29	906	1979
1	10	1	7	1	6	68	315
1	11	5	15	4	11	481	953
1	12	2	8	2	8	112	400
2	8	80	116	50	69	6861	8630
2	9	17	33	14	27	844	2672
2	10	15	23	13	20	818	1625
2	11	3	4	3	4	164	754
3	8	41	95	30	66	4847	8964
3	9	43	95	33	65	4600	8648
3	10	27	51	25	41	2122	3572

The table shows the sample size of municipalities stratified by school-grade at baseline and the number of years between baseline and follow-up assessments. *N* cohorts indicates the number of municipality-year cohorts, *N* unique municipalities indicates the number of unique municipalities the cohorts came from. Note that municipalities can be counted multiple times for different T1 grade-levels and time-intervals

### Analyses

**Fig. 1:**
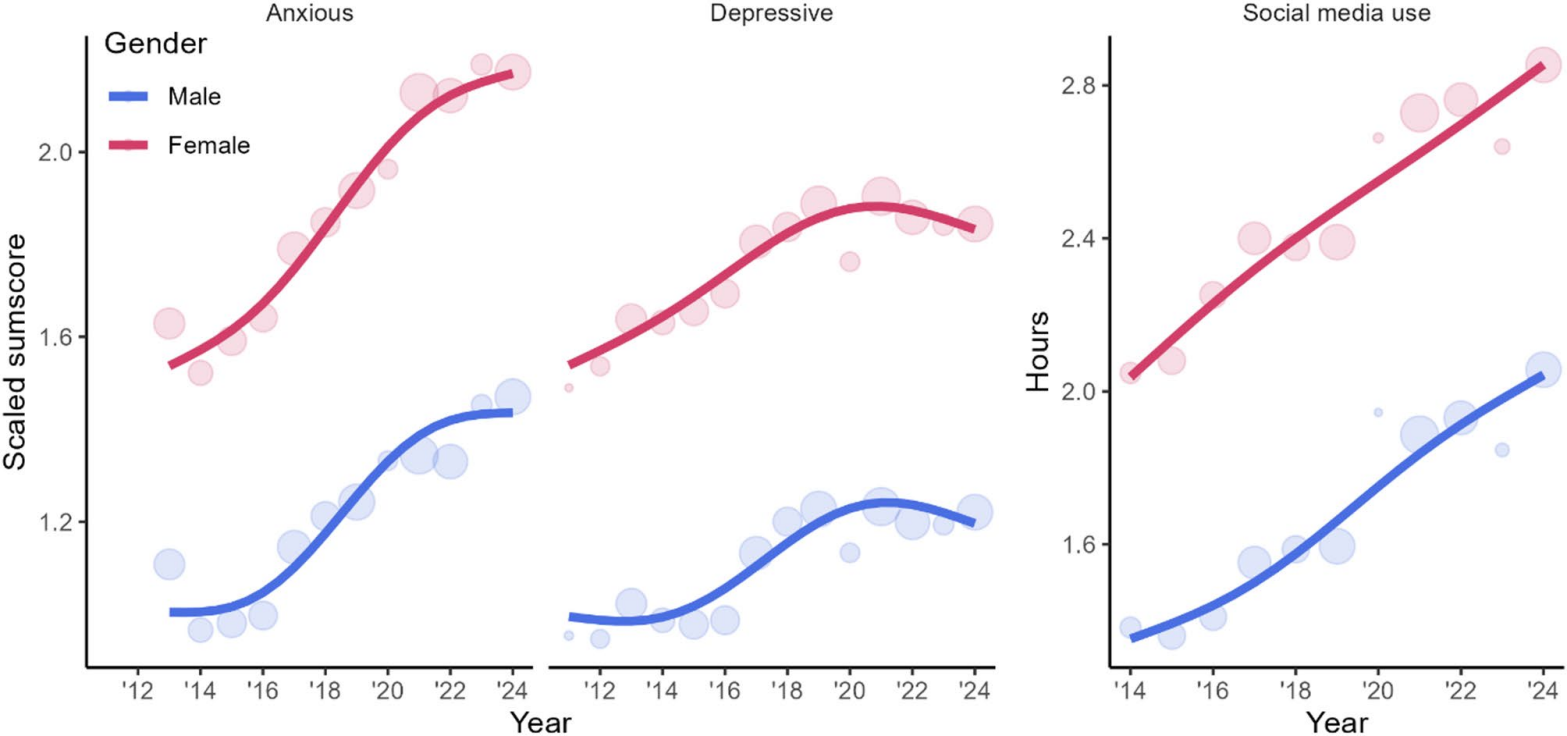
Time trends in outcomes and exposure Time trends for scaled sum scores of anxiety, depressive symptoms, and social media use, split by gender, for the entire Ungdata sample. Values for outcomes are scaled to allow for easy comparisons of the trends. Scaling is performed using data from 2013 to 2015. For these years, the mean for boys is 1, the standard deviation of sum scores is 1, and the mean score for girls is offset by the raw mean difference divided by the pooled standard deviation. This scaling facilitates a comparison of the time trends of the outcomes while maintaining the difference between boys and girls. Points indicate average values for a year, whereas the size of the points indicates the sample size. Lines are trend-lines estimated from the data

Descriptive time trends for social media use and anxious and depressive symptoms were estimated separately for boys and girls using the entire Ungdata dataset (see Fig. 1). The data were scaled using 2013–2015 data to facilitate a comparison of time trends for anxiety and depressive symptoms while maintaining the difference between boys and girls.

To estimate the municipality-level effects of social media use on self-reported symptoms of anxiety and depression, we employed a Bayesian multilevel model using the R package *brms* [28], which utilizes the Stan probabilistic programming language and a Hamiltonian Monte Carlo sampler for model estimation [29]. The analysis was designed to approximate a pre-test–post-test design, adjusting for both pre-treatment time-varying and time-constant confounders. We ran two analyses, one with depressive symptoms as the outcome and one with anxiety symptoms as the outcome.

To address time-varying confounding, we adjusted for baseline symptoms of the respective outcome, family socioeconomic status, sex, birth year, and the time interval between baseline and follow-up. To account for time-invariant confounders, we used a combination of fixed and random effects. Random effects were specified at multiple levels to account for clustering and time-constant confounding at the municipality level [30]. Specifically, we included municipality-level random effects to adjust for time-invariant contextual differences, and nested random effects by school year, time interval, and birth year to account for repeated observations within municipalities and variations across cohorts. We adjusted for potential bias from random effects components by including municipality-level averages of exposure (SMU) at baseline [31]. Urbanicity of municipalities was accounted for by an urbanicity index provided by Statistics Norway [27]. Given the potential impact of COVID-19-related disruptions in 2021, we included an indicator variable for this period. Exposure random slopes were employed to allow the effects of SMU to vary across key covariates such as age and time between the first and second measurement.

To minimize parametric assumptions about the functional form of the exposure-outcome association, we used spline functions to model continuous covariates, including socioeconomic status and social media use. Model residual variance was allowed to vary by sex and birth year. See page 8 of the Supplementary materials for a complete model specification.

To estimate average treatment effects (ATEs) of social media use on mental health outcomes, we applied G-estimation methods [32]. Altogether, this approach ensures a robust estimation of social media’s impact on adolescent mental health while addressing confounding at multiple levels through pre-test adjustment, hierarchical modeling, and flexible functional forms.

## Results

Girls reported more internalizing problems and more social media use than boys, and both groups generally reported higher scores on all measures at Time 2 than Time 1. There was a roughly equal proportion of girls and boys in the sample (Table 2).

**Table 2:** Descriptive statistics of the sample

Outcome	Time	Variable	Boys	Girls	% girls
Anxiety	1	Social media use	1.26 (0.28)	2.14 (0.31)	50
	1	Anxiety	0.21 (0.08)	0.59 (0.17)	50
	1	Ses	2.14 (0.12)	2.14 (0.12)	50
	2	Social media use	1.65 (0.23)	2.50 (0.23)	49
	2	Anxiety	0.33 (0.08)	0.84 (0.14)	49
	2	Ses	2.05 (0.16)	2.07 (0.15)	49
Depression	1	Social media use	1.40 (0.34)	2.32 (0.36)	52
	1	Depression	0.68 (0.14)	1.18 (0.22)	52
	1	Ses	2.10 (0.15)	2.14 (0.14)	52
	2	Social media use	1.81 (0.31)	2.64 (0.28)	52
	2	Depression	0.86 (0.12)	1.38 (0.15)	52
	2	Ses	2.01 (0.17)	2.06 (0.18)	52

The table shows mean scores and standard deviations (over municipalities) for social media use, outcomes and socioeconomic status (Ses) for the analysis sample, stratified by outcome scale (anxiety and depression symptoms), measurement point (1 = Baseline, 2 = Follow-up), and gender

The data show a steady increase in anxiety, depressive symptoms, and social media use, with depressive symptoms stabilizing in recent years (Fig. 1). Girls consistently reported more symptoms, and more time spent on social media than boys.

**Fig. 2:**
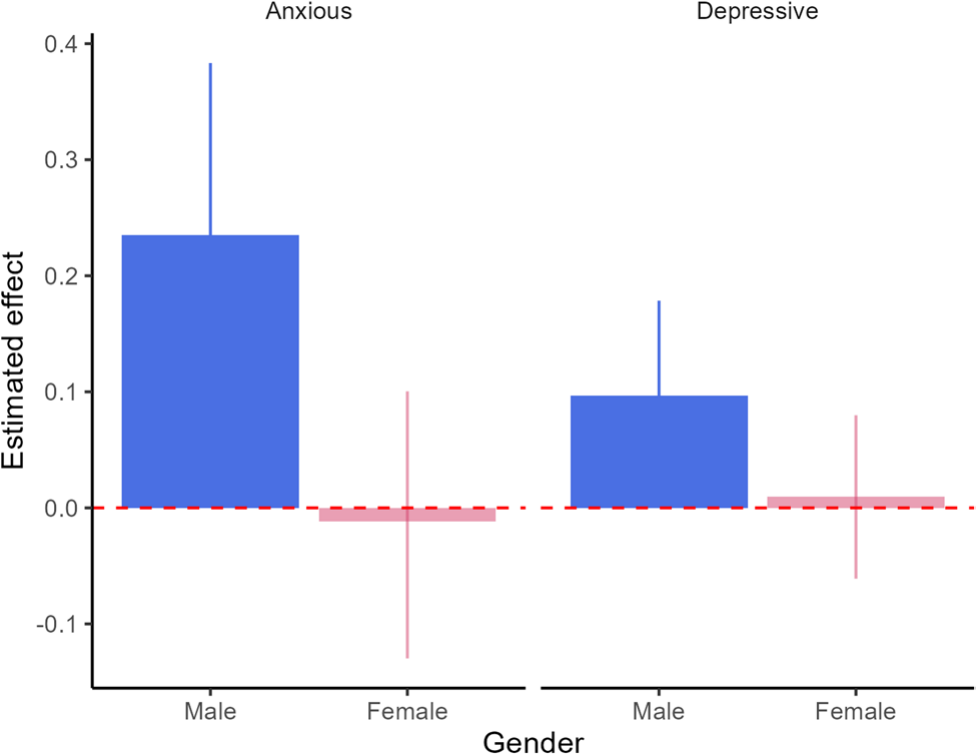
Effect of SMU on anxiety and depression Estimated standardized effect of SMU at Time 1 on mental health outcomes at Time 2. The effects are standardized to enable comparison across outcome and gender, and indicate how many standard deviations the outcome increases when SMU increases by one standard deviation. Each column represents anxiety and depressive symptoms outcomes for boys (blue) and girls (red), respectively. The height of the bars indicates the strength and direction of the effect estimate, while the vertical lines denote 95% credibility intervals. Effect estimates where the credibility interval includes 0 are shown in a lighter color

One standard deviation increase in social media use was not clearly associated with a change in anxiety scores for girls (−0.01 [−0.13, 0.10]) and was associated with an increase of 0.24 [0.09, 0.38] in anxiety scores for boys. Similarly for depressive symptoms, an increase of one standard deviation of social media use was not clearly associated with a change in depression scores for girls (0.01 [−0.06, 0.08]) and was weakly associated with an increase of 0.10 [0.01, 0.18] in depression scores for boys (Fig. 2). There were some variations in effects depending on the age at T1, but no consistent pattern was apparent (see Figures S8 and S15 in Supplementary materials). Effects for boys were slightly larger for a 1-year interval between T1 and T2 compared with longer time-intervals (see Figures S9 and S16 in Supplementary materials).

The results are summarized in Table 3 as standardized and unstandardized effect estimates, showing small effect sizes for boys and negligible effect size for girls. The unstandardized results give the mental health effects of a 1-hour increase in a cohorts’ average social media use, which is arguably a substantial increase. The models explained a substantial amount of variation in the T2 outcomes, with *r*^*2*^ = 0.6 − 0.8.

**Table 3:** Unstandardized and standardized results

Gender	Standardized	Anxiety estimate (95% CI)	Depression estimate (95% CI)
Boys	-	0.09 (0.03, 0.15)	0.04 (0.01, 0.07)
Girls	-	0.00 (−0.05, 0.04)	0.00 (−0.03, 0.03)
Boys	xy	0.24 (0.09, 0.38)	0.10 (0.01, 0.18)
Girls	xy	−0.01 (−0.13, 0.10)	0.01 (−0.06, 0.08)
Boys	y	0.70 (0.26, 1.14)	0.25 (0.03, 0.46)
Girls	y	−0.03 (−0.31, 0.24)	0.02 (−0.15, 0.19)

The table shows the estimated effect of social media use on anxious and depressive symptoms for boys and girls, respectively. Xy standardization refers to the expected increase in the outcome in standard deviations from a one standard deviation increase in social media use. Y standardization refers to the expected increase in standard deviations of the outcome from a one hour increase in social media use

## Discussion

This large-scale study of Norwegian adolescents’ internalizing problems indicates that social media use on the municipality level may have some effects, but these are not consistent across population groups or outcomes. Specifically, we estimated small effects on subsequent anxiety and depression among boys, but not among girls. Our findings largely parallel those from individual-level studies showing that SMU and mental health complaints are generally correlated within cohorts [7], but where a recent meta-analysis of experimental studies found no consistent evidence of a causal relationship [5].

Confronted with deteriorating mental health among youths in large parts of the world, it is imperative that researchers make use of available large-scale data to identify its causes [1, 2]. Any claim to estimate causal effects from observational data rests on the assumption of no unmeasured confounding [21]. We accounted for all time-varying unobserved confounding prior to the assessment of SMU by adjusting for the pre-treatment levels of internalizing problems, and we adjusted for time-constant confounders through a mixture of fixed and random effects [31]. The large proportion of variance in internalizing symptoms explained by our models supports our assumption that we have not left out key explanatory variables. Still, a crucial insight from the causal inference literature is that sufficient confounder adjustment cannot be verified empirically, but relies instead on subject matter knowledge and debate [21]. One potential source of bias unique to aggregate-level research is migration between municipalities across assessment waves [17], but we know of no evidence of substantial migration patterns in Norway during the study period. Taken together, the combination of a solid study design that allows adjusting for many unobserved confounders and the finding that the analysis models explain a large portion of the variance make a causal interpretation of our results plausible.

We estimated a small effect of SMU on anxiety and depression for boys, which was somewhat larger for a 1-year interval between assessments compared to longer intervals. Still, we are hesitant to overinterpret this on two accounts. Firstly, the overall standardized effect size was quite small. Researchers in the social media field disagree widely over what to consider a meaningful effect [7]. Our model suggests that a mean increase of 1 h of SMU corresponds to a 0.09-point increase in anxiety symptoms and a 0.04-point increase in depressive symptoms on a scale from 1 to 4. We would argue that the effect on depression is negligible while the effect on anxiety is potentially meaningful if replicated. Secondly, the gendered pattern is opposite from what is commonly found, namely that there is a stronger association between SMU and internalizing problems in girls [15, 16, 33]. Girls typically spend more time on social media and report more internalizing symptoms than their male peers [34], also in our data. It is not readily apparent why boys should be more susceptible to plausible group or individual-level mechanisms for mental health effects of SMU, such as social comparisons or contagion of negative emotions [34–36]. For these reasons, Keyes & Platt [34] have argued that any plausible cause for the increase in youth internalizing problems should display a gendered effect which is stronger for girls than for boys. We are thus inclined to interpret the overall result of our study as an inconsistent effect of SMU on youth internalizing symptoms at the municipality level.

A key contribution of our study is that the design establishes the direction of effects—from social media use to subsequent mental health—overcoming a central limitation of much prior cross-sectional research [37]. This also expands on more recent work by Nilsen and colleagues [16], who found that increases in a municipality’s average social media use (SMU) were associated with concurrent increases in physical health complaints, particularly among girls. To the extent that physical health complaints and internalizing symptoms are related [38], our contrasting findings could suggest that the concurrent associations reflect broader societal changes or reverse causality.

The aggregated level of analysis in this study has both strengths and limitations. It is well suited to identify indirect network or cultural effects of living in a peer group with high social media use and can provide insight into the potential impact of municipality-wide initiatives aimed at reducing access to social media. However, drawing conclusions about individual-level effects from aggregated data requires stronger assumptions than we are willing to make [18]. It remains possible that social media use affects youth mental health at the individual level within municipalities. For instance, if social media’s impact is highly non-linear—primarily affecting extreme users, as some studies suggest—individual-level effects may be obscured in aggregate data [39–41]. It is also possible that SMU disproportionately affects more vulnerable youth [42], which at the level of municipalities can be balanced out by youth who gain something from social media [43]. A similar heterogeneity may arise from different patterns of use: for example, passive and public use (e.g., scrolling through feeds) may have negative effects, whereas active and private use (e.g., messaging friends) may have positive effects, although the evidence for these distinctions remains mixed [9, 10]. Therefore, our findings do not justify a broad recommendation for parents or professionals to disregard concerns about individual youth’s social media use. The results are congruent with a perspective that social media has both negative and positive effects on individuals, which may balance each other out at the population level [7, 37, 43].

It is important to recognize that our analysis does not preclude other worrying group-level effects of SMU, such as polarization of gender roles or political opinions [44]. It is also possible that the mere presence of social media in a community is enough to have downstream effects on mental health beyond the average time spent on social media, which could explain the discrepancy between our findings and the detrimental effects found from the introduction of Facebook to US colleges [15]. However, the decline in Norwegian youth mental health began prior to the widespread adoption of smartphones and social media, indicating that these factors cannot be the sole drivers of the trend [1].

One implication of our findings is that municipality-wide interventions to reduce the overall SMU among youth may not improve the population’s internalizing problems. This is in accordance with the mixed findings on the effects of school smartphone bans [19, 20, 45]. One recent UK study showed no differences in mental well-being among pupils in schools with restrictive or permissive phone policies [20]. However, the same study also found no differences in the overall time spent on social media between groups, suggesting that school phone bans in their current form are ineffective at reducing adolescent SMU [20]. Interventions targeting vulnerable youth with heavy social media use have shown more promising effects [46], congruent with a perspective that SMU can be harmful to some even if no effect is found on the population level.

### Limitations

The study’s strengths include a large, nation-wide dataset of Norwegian adolescents and a causally informed analysis. However, some limitations should be noted. We assessed the average time youths reported spending on social media, with the upper category being 3h or more. It is possible that this is too narrow a range, as it hinders us from differentiating youths using social media 3.5 h a day from more than 6 h a day [4, 47]. Additionally, assessing screen time more broadly, as well as using more nuanced measures of how young people spend their time on social media (active versus passive use; type of platform; motivation and purpose behind SMU) could potentially shed light on important mental health effects beyond what we were able to in the present study. Finally, the data were obtained from students during school hours. While this allowed for assessing a large number of youths with minimal distractions, it also limits our analyses to youths who were present at school on the day of data collection. It is possible that students who were absent from school represent a more vulnerable group and would show a different effect from SMU.

## Conclusions

Accounting for both observed and unobserved confounders, the present study indicates that, at the municipality level, increases in social media use may exert a modest negative effect on boys’ mental health. However, social media use does not appear to constitute a primary driver of the recent rise in depression and anxiety among adolescents, which has been documented predominantly among girls. The study is congruent with a perspective that social media may have both detrimental and positive effects on youth mental health which outweigh each other at the population level.

## Supplementary Information

Supplementary Material 1

Supplementary Material 2

## Data Availability

The full Ungdata survey used in the study can be downloaded from https://www.ungdata.no/wp-content/uploads/2020/09/Ungdata-Dokumentasjonsrapport-2010-2019-PDF-1.pdf. The dataset analyzed in the current study can be downloaded from https://surveybanken.sikt.no/en/study/query/ungdata/page/1. Codes for Oslo city districts are not in the file but can be requested from the Ungdata team.
