# Supplementary material for: The effect of municipality-level social media use on youth mental health
Source: BMC Public Health. 2025 Oct 14;25:3472. doi: 10.1186/s12889-025-24727-4 (PMC12523118; doi:10.1186/s12889-025-24727-4)
Supplement: Supplementary file 1 — Supplementary Material 1 [file 12889_2025_24727_MOESM1_ESM.docx]

Survey items used in the paper The Effect of Municipality-Level Social Media Use on Youth Mental Health.

Social media

| Item | Response options |
| --- | --- |
| **Think about what you do on a normal day. How much time do you spend on the following things:** | 1: No time  2: Less than 30 minutes  3: 30 minutes – 1 hour  4: 1-2 hours  5: 2-3 hours  6: More than 3 hours |
| Social media (Facebook, Instagram, etc.) |  |

Depressive symptoms

Items were adapted from the Depressive Mood Inventory(1)

| Item | Response options |
| --- | --- |
| **During the past week, have you been affected by any of the following issues:** | 1: Not been affected at all  2: Not been affected much  3: Been affected quite a lot  4: Been affected a great deal |
| Felt that everything is a struggle |  |
| Had sleep problems |  |
| Felt unhappy, sad or depressed |  |
| Felt hopelessness about the future |  |
| Felt stiff or tense |  |
| Worried too much about things |  |

Anxiety symptoms

Items were adapted from the Hopkins Symptom Checklist(2)

| Item | Response options |
| --- | --- |
| **During the past week, have you been affected by any of the following issues:** | 1: Not been affected at all  2: Not been affected much  3: Been affected quite a lot  4: Been affected a great deal |
| Suddenly felt scared for no reason |  |
| Felt constant fear or anxiety |  |
| Been nervous or felt uneasy |  |

Socioeconomic status

Socioeconomic status was provided as a continuous scale from 0 to 3 by Ungdata, calculated using the following items:

| Item | Response options |
| --- | --- |
| Did your father and mother go to university or to a university college? Select one answer for each parent. If you are not in touch with one or both of your parents, then skip the question about that parent. |  |
| Father | 1: Yes  2: No |
| Mother | 1: Yes  2: No |
| How many books do you think are in your family home? | 1: No books  2: Less than 20 books  3: 20-50 books  3: 100-500 books  4: 500-1000 books  4: More than 1000 books |
| Does your family have a car? | 1: No  2: Yes, one  3: Yes, two or more |
| Do you have your own bedroom? | 1: Yes  2: No |
| How many times have you travelled somewhere on holiday with your family over the past year? | 1: Never  2: Once  3: Twice  4: More than twice |
| How many computers or tablet computers does your family have? | 1: None  2: One  3: Two  4: More than two |

References

1. Kandel DB, Davies M. Epidemiology of depressive mood in adolescents: An empirical study. Archives of General Psychiatry. 1982;39(10):1205-12.

2. Derogatis LR, Lipman RS, Rickels K, Uhlenhuth EH, Covi L. The Hopkins Symptom Checklist (HSCL): A self‐report symptom inventory. Behavioral Science. 1974;19(1):1-15.

The complete Ungdata survey can be found at <https://www.ungdata.no/wp-content/uploads/2020/09/Ungdata-Dokumentasjonsrapport-2010-2019-PDF-1.pdf>.
