# Supplementary material for: The effect of municipality-level social media use on youth mental health
Source: BMC Public Health. 2025 Oct 14;25:3472. doi: 10.1186/s12889-025-24727-4 (PMC12523118; doi:10.1186/s12889-025-24727-4)
Supplement: Supplementary file 2 — Supplementary Material 2 [file 12889_2025_24727_MOESM2_ESM.docx]

Supplementary materials

## General description of the statistical model

To estimate the municipality-level effects of social media use on self-reported symptoms of anxiety and depression, we employed a Bayesian multilevel model using the R package brms (Bürkner 2017), which utilizes the Stan probabilistic programming language and a Hamiltonian Monte Carlo sampler for model estimation (Carpenter et al. 2017). The analysis was designed to approximate a pre-test–post-test framework, adjusting for both time-varying and time-constant confounders.

The primary outcome represents symptoms of anxiety or depression at follow-up, while social media use serves as the exposure variable, measured at baseline. To control for potential confounding, we adjusted for key pre-test covariates, including baseline symptoms, family socioeconomic status, sex, school year, urbanicity of the municipality and the time interval between baseline and follow-up.

Random effects were specified at multiple levels to account for clustering and time-constant confounding at the municipality level (Gelman and Hill 2006). Specifically, we included:

- Municipality-level random effects to adjust for time-invariant contextual differences.
- Nested random effects by school year, time interval, and birth year to account for repeated observations within municipalities and variations across cohorts.
- Exposure random slopes to allow the effects of social media use to vary across key covariates.
- To minimize parametric assumptions about the functional form of the exposure-outcome association, we incorporated penalized spline functions for continuous covariates, including socioeconomic status and social media use.
- Additionally, we adjusted for potential bias from random effects components by including municipality-level averages of exposure baseline (Neuhaus and Kalbfleisch 1998; Mundlak 1978).

Given the potential impact of COVID-19-related disruptions in 2021, we included an indicator variable for this period and modeled its interaction effects. Model residual variance was allowed to vary by sex and birth year.

To estimate average treatment effects (ATEs) of social media use on mental health outcomes, we applied G-estimation methods (Robins 1986). The model assumes a Gaussian likelihood, appropriate given the continuous nature of the outcome variables.

This approach ensures a robust estimation of social media’s impact on adolescent mental health while addressing confounding at multiple levels through pre-test adjustment, hierarchical modeling, and flexible functional forms.

## The effect of social media use on anxiety

This analysis investigates the effect of social media use on anxiety.

We use number of hrs of social media use as exposure variable.

| 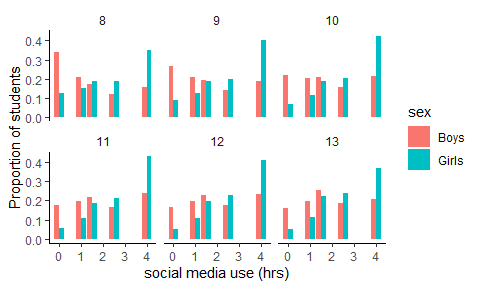  Figure S1: Distribution of social media use by school year and sex. |
| --- |

We use the mean-score of ratings on 3 anxiety items as outcome variable (see [Figure S1](#fig-outcomesocmed_hrsangst)). The outcome variable is measured 1-3 years after the exposure variable. **In the following, we use mean score as the outcome scale.**

| 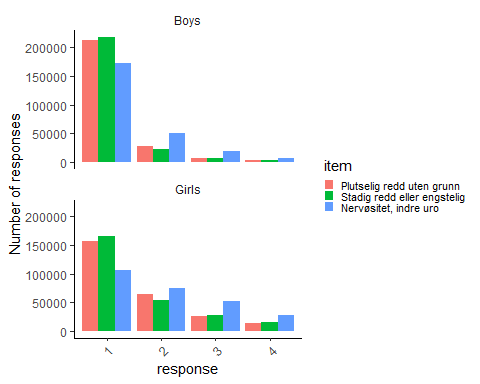  Figure S2: Distribution of anxiety symptoms by item and sex |
| --- |

Chronbach’s alpha for the anxiety scale is 0.87.

### Detailed analysis setup

For the current analysis, we are using time of social media use and mean score of anxiety as exposure and outcome variables, respectively. Because our level of analysis is municipalities, we use individual student-level scores to calculate municipality-level means, which are then used for the statistical analysis.

This analysis includes data from 262 units, i.e. cohorts within municipalities. The distribution of units according to urbanicity from 1 highest to 6 lowest is 1, 69, 75, 58, 48, 11. Unit level data were calculated from 22,242 individual students. [Table S1](#tbl-samplesize1) shows the number of cohorts as well as the total number of pupils included in the study stratified by other characteristics.

The distribution of exposure and outcome variables is shown in [Figure S3](#fig-distcountysocmed_hrsangst), which also shows that there is a noticeable variation of average social media use between municipalities. Mean and standard deviations of exposure and outcome are shown in [Table S3](#tbl-basicstats1)

| 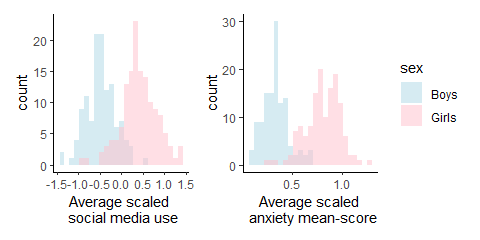  Figure S3: Distribution of averge social media use & anxiety in counties by sex. |
| --- |
| Table S1: Sample size (cohorts within municipalities) and number of unique municipalities for exposure social media use and outcome anxiety by years from t_1_ to t_2_ and school year of the first measurement (t_1_).   \| Years t1 to t2 \| School year t1 \| N \| N unique municipalities \| N_students \| \| --- \| --- \| --- \| --- \| --- \| \| 1 \| 8 \| 11 \| 6 \| 419 \| \| 1 \| 9 \| 17 \| 10 \| 906 \| \| 1 \| 10 \| 1 \| 1 \| 68 \| \| 1 \| 11 \| 5 \| 4 \| 481 \| \| 1 \| 12 \| 2 \| 2 \| 112 \| \| 2 \| 8 \| 80 \| 50 \| 6861 \| \| 2 \| 9 \| 17 \| 14 \| 844 \| \| 2 \| 10 \| 15 \| 13 \| 818 \| \| 2 \| 11 \| 3 \| 3 \| 164 \| \| 3 \| 8 \| 41 \| 30 \| 4847 \| \| 3 \| 9 \| 43 \| 33 \| 4600 \| \| 3 \| 10 \| 27 \| 25 \| 2122 \| |

| Table S2: Sample size (cohorts within municipalities) by year of the first measurement (t_1_) for social media use and outcome anxiety . Percent kept is the proportion of all municipalities with t_1_ and t_2_ data in the study sample, which could be included because the sample size between both measurement time points deviated by less than 15%.   \|  \| 2014 \| 2015 \| 2016 \| 2017 \| 2018 \| 2019 \| 2020 \| 2021 \| 2022 \| \| --- \| --- \| --- \| --- \| --- \| --- \| --- \| --- \| --- \| --- \| \| N municiaplities \| 33 \| 49 \| 79 \| 51 \| 30 \| 1 \| 13 \| 4 \| 2 \| \| Percent kept \| 15 \| 14 \| 23 \| 21 \| 20 \| 1 \| 20 \| 13 \| 11 \| |
| --- | --- | --- | --- | --- | --- | --- | --- | --- | --- | --- | --- | --- | --- | --- | --- | --- | --- | --- | --- | --- | --- | --- | --- | --- | --- | --- | --- | --- | --- | --- |

| Table S3: Mean and standard deviations (over municipalities) for social media use and outcome anxiety and sosioeconomic status (ses), stratified by sex and measurement time point.   \| variable \| time \| Boy \| Girl \| Percent Girls \| \| --- \| --- \| --- \| --- \| --- \| \| social media use \| 1 \| 1.26 (0.28) \| 2.14 (0.31) \| 50 \| \| anxiety \| 1 \| 0.21 (0.08) \| 0.59 (0.17) \| 50 \| \| ses \| 1 \| 2.14 (0.12) \| 2.14 (0.12) \| 50 \| \| social media use \| 2 \| 1.65 (0.23) \| 2.50 (0.23) \| 49 \| \| anxiety \| 2 \| 0.33 (0.08) \| 0.84 (0.14) \| 49 \| \| ses \| 2 \| 2.05 (0.16) \| 2.07 (0.15) \| 49 \| |
| --- | --- | --- | --- | --- | --- | --- | --- | --- | --- | --- | --- | --- | --- | --- | --- | --- | --- | --- | --- | --- | --- | --- | --- | --- | --- | --- | --- | --- | --- | --- | --- | --- | --- | --- | --- |

### Exploratory plots

We re-structure the data to plot, as shown in [Figure S4](#fig-scattersocmed_hrsangst), for each cohort/municipality unit social media use timepoint 1 ($t_{1}$) against anxiety mean-scores one year later ($t_{2}$). [Figure S4](#fig-scattersocmed_hrsangst) A is misleading, as the correlation is mainly driven by the sex differences. [Figure S4](#fig-scattersocmed_hrsangst) B shows the association after removal of sex difference in social media use and depression mean-scores.

| 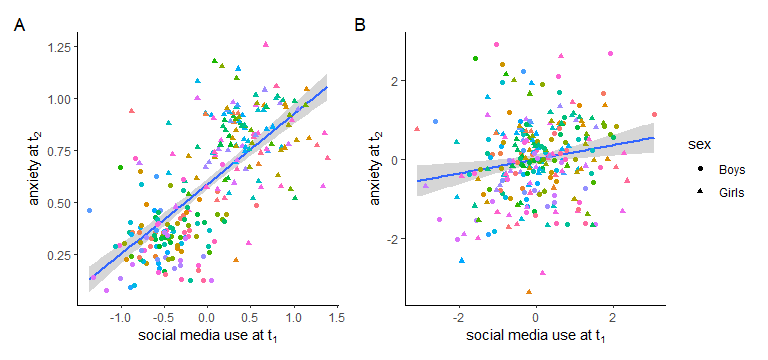  Figure S4: Scatter plot of social media use (t_1_) versus anxiety mean score (t_2_) by sex. Colors indicate municipality. A: raw data, B: standardized by sex. Each point is one unit in the analysis and represents individuals from the same cohort and municipality. |
| --- |
| 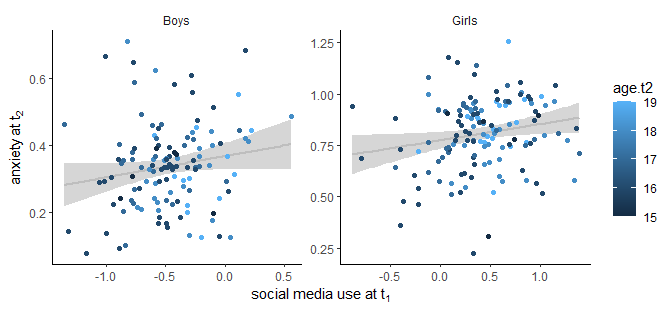  Figure S5: Scatter plot of anxiety (t_1_) versus anxiety mean score (t_2_) by sex. Colors indicate age at t_2_. Lighter color in the top right corner of a panel indicates an age effect. |
| 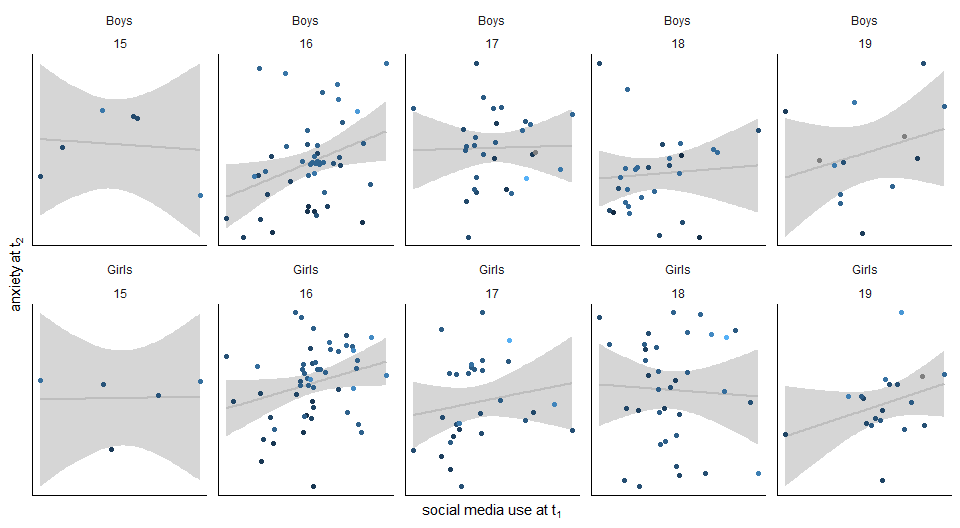  Figure S6: Scatter plot of social media use use (t_1_) versus anxiety mean score (t_2_) by sex. Colors indicate age at t_2_. Lighter color in the top right corner indicate a cohort effect. |

### Statistical Modeling Approach

To estimate the **municipality-level effects of social media use on self-reported symptoms of anxiety and depression**, we employed a **Bayesian multilevel model** using the R package **brms** (Bürkner 2017), which utilizes the **Stan** probabilistic programming language and a **Hamiltonian Monte Carlo sampler** for model estimation (Carpenter et al. 2017). The model was designed to approximate a **pre-test–post-test** framework, adjusting for both **time-varying** and **time-constant confounders** to better estimate the causal effects of social media use.

#### **Outcome and Exposure Variables**

The **outcome variable** (m_t2_y) represents symptoms of **angst** at **follow-up** (t2), while the **exposure variable** (m_t1_x) captures **socmed_hrs** at **baseline** (t1). The model accounts for pre-test differences by adjusting for the **baseline mental health status** (m_t1_y), thus mitigating confounding from pre-existing factors.

#### **Key Covariates and Adjustment Variables**

The model controls for several covariates known to influence both social media use and mental health outcomes, including:

- **Family socioeconomic status at baseline** (m_t1_SES)
- **Sex** (sex)
- **School year at baseline** (school.year.t1)
- **Time interval between baseline and follow-up** (delta_t)

Additionally, we included adjustments for potential **bias introduced by random effects components** (Neuhaus and Kalbfleisch 1998, mundlak1978) by adding municipality-level averages of exposure which help control for cluster-level confounding.

#### **Random Effects Structure**

To **account for clustering** within municipalities and schools, and to capture variations in exposure effects, we included several hierarchical random effects:

- **Municipality-level random intercept** (1 | kommune) to adjust for **time-constant confounders** such as urbanicity.
- **Nested random effects by school year, time interval, and birth year** (1 | school.year.t2:delta.t:byear) to capture cohort-based differences in mental health trends.
- **Random slopes for exposure by school year, time interval, and sex** (0 + m_t1_x | school.year.t1:delta.t:sex), allowing the association between social media use and mental health to vary by demographic and temporal factors.

#### **Flexible Modeling of Nonlinear Relationships**

To avoid restrictive parametric assumptions, we employed **penalized spline functions** (s()) to model nonlinear associations for key continuous predictors:

- A **spline term for socioeconomic status at baseline** (s(m_t1_SES)), capturing potential nonlinear relationships between SES and mental health.
- A **multivariate spline for social media use and baseline mental health symptoms, stratified by sex** (s(m_t1_x, m_t1_y, by = sex)), allowing for sex-specific exposure-outcome relationships.

#### **Adjustment for COVID-19-Related Effects**

Given the potential impact of **pandemic-related disruptions** in **2021**, we included a binary indicator variable (is.2021) to adjust for differences in mental health trends during this period. Additionally, a random slope for the pandemic effect ((0 + is.2021 | sex:school.year.t2:byear)) accounts for variation across demographic subgroups.

#### **Residual Variance Structure**

The model allows **residual variance** (sigma) to vary by key demographic factors. Specifically, we model residual variance conditional on sex and birth year: sigma ~ sex + sbyear.

#### **Estimation of Treatment Effects**

To estimate the **average treatment effects (ATEs)** of social media use on mental health, we applied **G-estimation methods** (Robins 1986). The outcome was assumed to follow a **Gaussian likelihood**, appropriate given the continuous nature of the measured symptoms.

my_formula = bf(
 m_t2_y ~
 kavg_m_t1_x +
 (1|school.year.t2:delta.t:byear) +
 m_t1_kommunestørrelse +
 s(m_t1_SES) +
 is.2021 +
 s(m_t1_x, m_t1_y, by = sex) +
 (0 + m_t1_x | school.year.t1:delta.t:sex) +
 (1 | kommune),
 sigma ~ sex + sbyear,
 family = gaussian())

## Results

[Figure S7](#fig-fATEsocmed_hrsangst) shows average marginal effects as the slope of the colored regression line. The grey regression line shows the anadjusted association. Points show the expected depression mean score at time point 2 at different levels of social media usage.

The most important results can be summarized as follows: Across different school classes and number of years between the first and second measurement, and across 262 cohorts from 113 unique municipalities (data from 2014-2024, each municipality can contribute with multiple data points) an increase of one hrs social media use was not clearly associated with change in points anxiety for girls (-0.00 (-0.05, 0.04)) and weakly associated with an increase of 0.09 (0.03, 0.15) points anxiety for boys. [Table S4](#tbl-tATE1) shows standardized and non-standardized effect estimates.

| 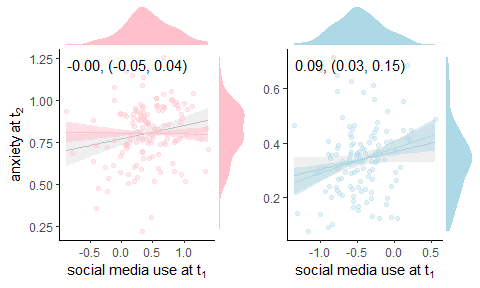  Figure S7: Effect of social media use on anxiety mean score. Each dot represents a cohort within a municipality that participated two times in Ungdata. The text indicates by how many units anxiety changed when social media use changed by one unit. Credible intervals are in parentheses. The effect size is visually represented by the slope of the colored regression lines. The grey slope visualises the effect size from an unadjusted model. The marginal distributions show observed distributions of social media use (top) and anxiety (right). The longer the vertical line in the right distribution is, the stronger is the effect of the social media use on anxiety . |
| --- |
| Table S4: Estimated effects of social media use on anxiety with credible intervals. Unstandardized, xy-standardized and y-standardized.   \| sex \| estimate \| standard \| \| --- \| --- \| --- \| \| Boys \| 0.09 (0.03, 0.15) \| - \| \| Girls \| -0.00 (-0.05, 0.04) \| - \| \| Boys \| 0.24 (0.09, 0.38) \| xy \| \| Girls \| -0.01 (-0.13, 0.10) \| xy \| \| Boys \| 0.70 (0.26, 1.14) \| y \| \| Girls \| -0.03 (-0.31, 0.24) \| y \| |
| 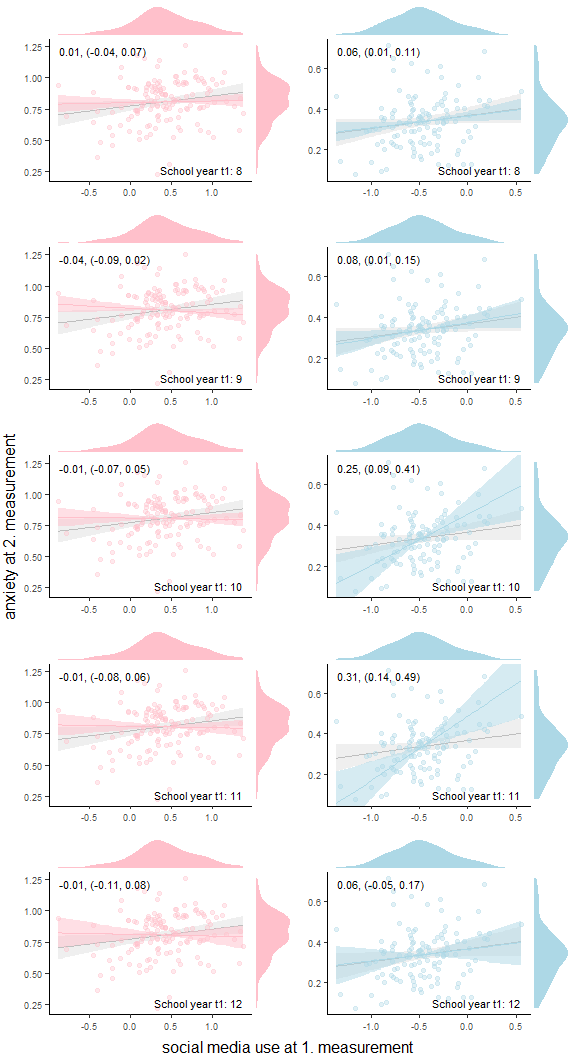  Figure S8: Marginal effects of social media use on anxiety by age group at first measurement |
| 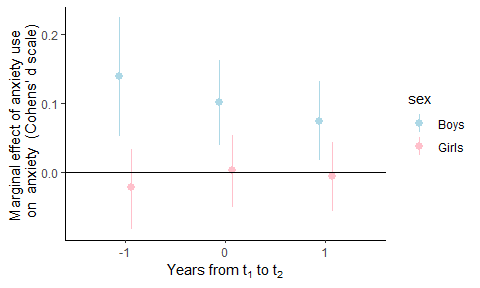  Figure S9: Marginal effects of social media use on anxiety by number of years between t_1_. |
| 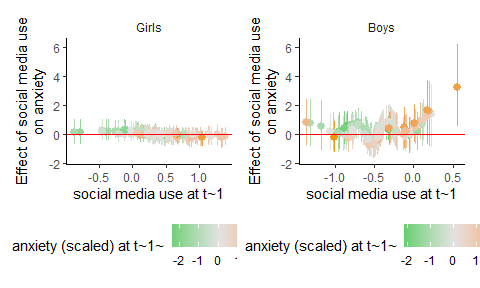  Figure S10: Estimated conditional treatment effect of social media use on anxiety by social media use at t_1_, stratified by sex. Colors indicate anxiety at t_1_ |

## The effect of social media use on depression

This analysis investigates the effect of social media use on depression.

We use number of hrs of social media use as exposure variable.

| 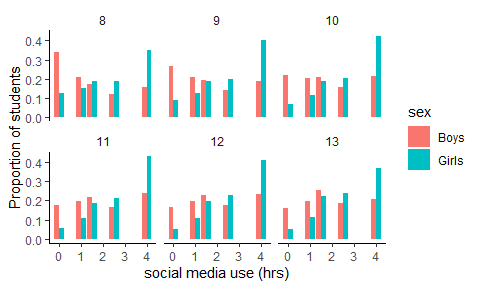  Figure S11: Distribution of social media use by school year and sex. |
| --- |

We use the mean-score of ratings on 6 depression items as outcome variable (see [Figure S11](#fig-outcomesocmed_hrsdepr)). The outcome variable is measured 1-3 years after the exposure variable. **In the following, we use mean score as the outcome scale.**

| 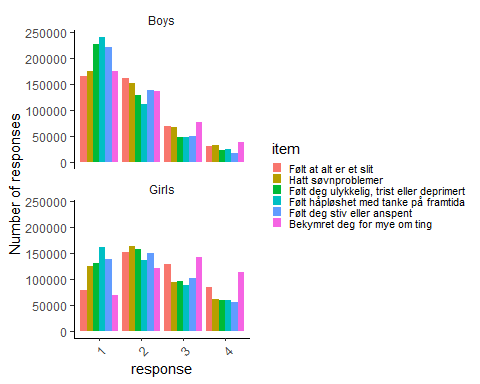  Figure S12: Distribution of depression symptoms by item and sex |
| --- |

Chronbach’s alpha for the depression scale is 0.89.

### Detailed analysis setup

For the current analysis, we are using time of social media use and mean score of depression as exposure and outcome variables, respectively. Because our level of analysis is municipalities, we use individual student-level scores to calculate municipality-level means, which are then used for the statistical analysis.

This analysis includes data from 528 units, i.e. cohorts within municipalities. The distribution of units according to urbanicity from 1 highest to 6 lowest is 1, 117, 169, 118, 80, 43. Unit level data were calculated from 40,014 individual students. [Table S5](#tbl-samplesize2) shows the number of cohorts as well as the total number of pupils included in the study stratified by other characteristics.

The distribution of exposure and outcome variables is shown in [Figure S13](#fig-distcountysocmed_hrsdepr), which also shows that there is a noticeable variation of average social media use between municipalities. Mean and standard deviations of exposure and outcome are shown in [Table S7](#tbl-basicstats2)

| 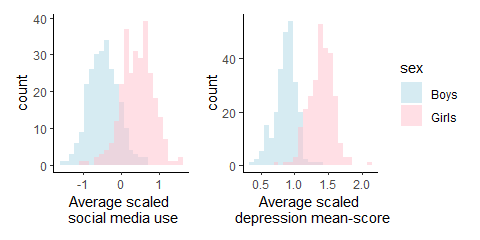  Figure S13: Distribution of average social media use & depression in counties by sex. |
| --- |
| Table S5: Sample size (cohorts within municipalities) and number of unique municipalities for exposure social media use and outcome depression by years from t_1_ to t_2_ and school year of the first measurement (t_1_).   \| Years t1 to t2 \| School year t1 \| N \| N unique municipalities \| N_students \| \| --- \| --- \| --- \| --- \| --- \| \| 1 \| 8 \| 38 \| 24 \| 1502 \| \| 1 \| 9 \| 43 \| 29 \| 1979 \| \| 1 \| 10 \| 7 \| 6 \| 315 \| \| 1 \| 11 \| 15 \| 11 \| 953 \| \| 1 \| 12 \| 8 \| 8 \| 400 \| \| 2 \| 8 \| 116 \| 69 \| 8630 \| \| 2 \| 9 \| 33 \| 27 \| 2672 \| \| 2 \| 10 \| 23 \| 20 \| 1625 \| \| 2 \| 11 \| 4 \| 4 \| 754 \| \| 3 \| 8 \| 95 \| 66 \| 8964 \| \| 3 \| 9 \| 95 \| 65 \| 8648 \| \| 3 \| 10 \| 51 \| 41 \| 3572 \| |

| Table S6: Sample size (cohorts within municipalities) by year of the first measurement (t_1_) for social media use and outcome depression. Percent kept is the proportion of all municipalities with t_1_ and t_2_ data in the study sample, which could be included because the sample size between both measurement time points deviated by less than 15%.   \|  \| 2014 \| 2015 \| 2016 \| 2017 \| 2018 \| 2019 \| 2020 \| 2021 \| 2022 \| 2023 \| \| --- \| --- \| --- \| --- \| --- \| --- \| --- \| --- \| --- \| --- \| --- \| \| N municiaplities \| 32 \| 47 \| 81 \| 52 \| 72 \| 56 \| 65 \| 96 \| 11 \| 16 \| \| Percent kept \| 15 \| 13 \| 23 \| 18 \| 17 \| 13 \| 25 \| 15 \| 23 \| 41 \| |
| --- | --- | --- | --- | --- | --- | --- | --- | --- | --- | --- | --- | --- | --- | --- | --- | --- | --- | --- | --- | --- | --- | --- | --- | --- | --- | --- | --- | --- | --- | --- | --- | --- | --- |

| Table S7: Mean and standard deviations (over municipalities) for social media use and outcome depression and sosioeconomic status (ses), stratified by sex and measurement time point.   \| variable \| time \| Boy \| Girl \| Percent Girls \| \| --- \| --- \| --- \| --- \| --- \| \| social media use \| 1 \| 1.40 (0.34) \| 2.32 (0.36) \| 52 \| \| depression \| 1 \| 0.68 (0.14) \| 1.18 (0.22) \| 52 \| \| ses \| 1 \| 2.10 (0.15) \| 2.14 (0.14) \| 52 \| \| social media use \| 2 \| 1.81 (0.31) \| 2.64 (0.28) \| 52 \| \| depression \| 2 \| 0.86 (0.12) \| 1.38 (0.15) \| 52 \| \| ses \| 2 \| 2.01 (0.17) \| 2.06 (0.18) \| 52 \| |
| --- | --- | --- | --- | --- | --- | --- | --- | --- | --- | --- | --- | --- | --- | --- | --- | --- | --- | --- | --- | --- | --- | --- | --- | --- | --- | --- | --- | --- | --- | --- | --- | --- | --- | --- | --- |

## Results

The most important results can be summarized as follows: Across different school classes and number of years between the first and second measurement, and across 528 cohorts from 181 unique municipalities (data from 2014-2024, each municipality can contribute with multiple data points) an increase of one hrs social media use was not clearly associated with change in points depression for girls (0.00 (-0.03, 0.03)) and weakly associated with an increase of 0.04 (0.01, 0.07) points depression for boys. [Table S8](#tbl-tATE2) shows standardized and non-standardized effect estimates.

| 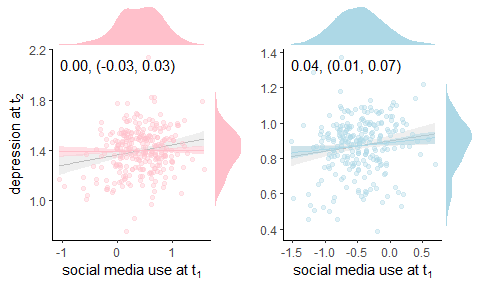  Figure S14: Effect of social media use on depression mean score. Each dot represents a cohort within a county that participated two times in Ungdata. The text indicates by how many units depression changed when social media use changed by one unit. Credible intervals are in parentheses. The effect size is visually represented by the slope of the colored regression lines. The grey slope visualises the effect size from an unadjusted model. The marginal distributions show observed distributions of social media use (top) and depression (right). The longer the vertical line in the right distribution is, the stronger is the effect of the social media use on depression . |
| --- |
| Table S8: Estimated effects of social media use on depression with credible intervals. Unstandardized, xy-standardized and y-standardized.   \| sex \| estimate \| standard \| \| --- \| --- \| --- \| \| Boys \| 0.04 (0.01, 0.07) \| - \| \| Girls \| 0.00 (-0.03, 0.03) \| - \| \| Boys \| 0.10 (0.01, 0.18) \| xy \| \| Girls \| 0.01 (-0.06, 0.08) \| xy \| \| Boys \| 0.25 (0.03, 0.46) \| y \| \| Girls \| 0.02 (-0.15, 0.19) \| y \| |
| 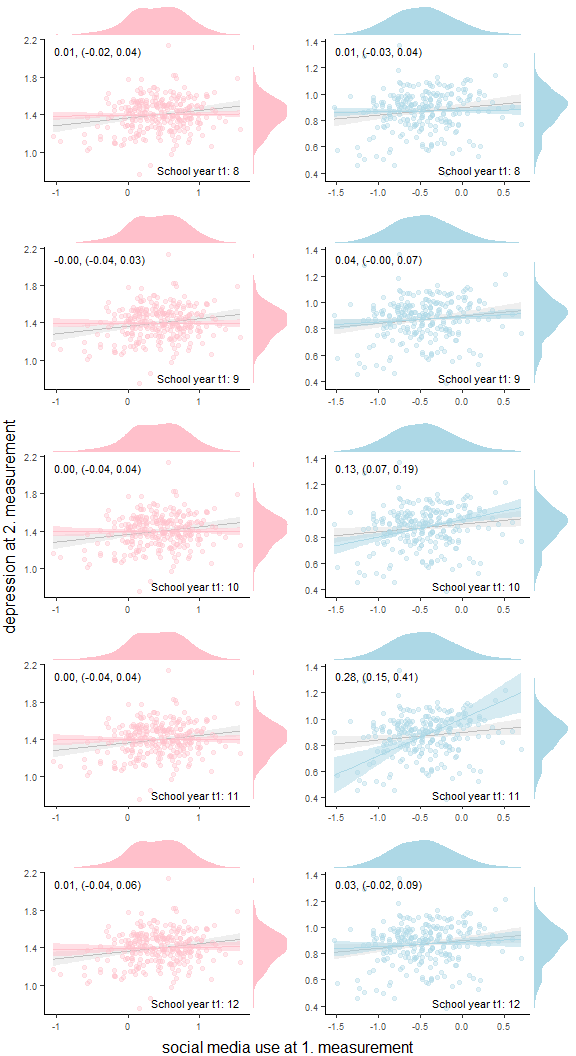  Figure S15: Marginal effects of social media use on depression by age group at first measurement |
| 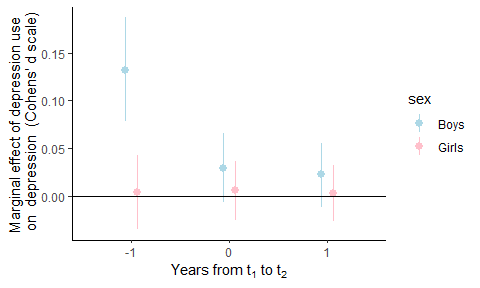  Figure S16: Marginal effects of social media use on depression by number of years between t_1_. |
| 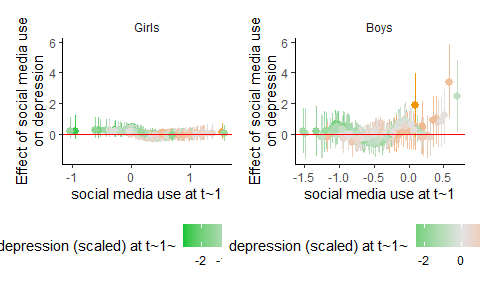  Figure S17: Estimated conditional treatment effect of social media use on depression by social media use at t_1_, stratified by sex. Colors indicate depression at t_1_ |

# Summary of Results

The following results are based on data from between 122 and 279 municipalities. Analyses that used anxiety as outcome—variables that Ungdata collected over fewer years—draw on fewer municipalities. On average, the models explained 81% of the variation in the outcome (min: 81%, max: 81%), suggesting that important unobserved confounding is typically unlikely.

For the outcome social media use an increase in time use for social media use med 0,5 hours is associated with in anxiety by 0.09 points (90% CI: 0.03, 0.15) among boys, while among girls the same increase in social media use is not linked to anxiety (-0.00 (90% CI: -0.05, 0.04)). Regarding depression, an increase in social media use is associated with in depression by 0.04 points (90% CI: 0.01, 0.07) among boys, while among girls is not linked to depression (0.00 (90% CI: -0.03, 0.03)).

| 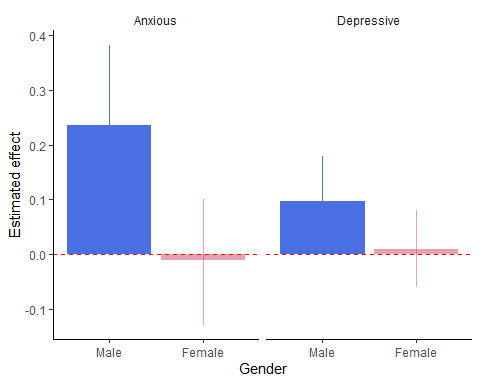  Figure S18: Estimated standardized effect of exposures on outcomes. The effects are standardized to enable comparison across different results and indicate how many standard deviations the outcome increases when the exposure increases by one standard deviation. Each row represents the effects for a specific exposure, and each column represents specific outcomes. The height of the bars indicates the strength and direction of the effect estimate, while the vertical lines denote 95% credibility intervals. Effect estimates where the credibility interval includes 0 are shown in a lighter color. Interpreting the estimated effects as causal effects requires the assumption of no unobserved confounding variables, which is not unlikely given the study design (see the text for details). |
| --- |
| 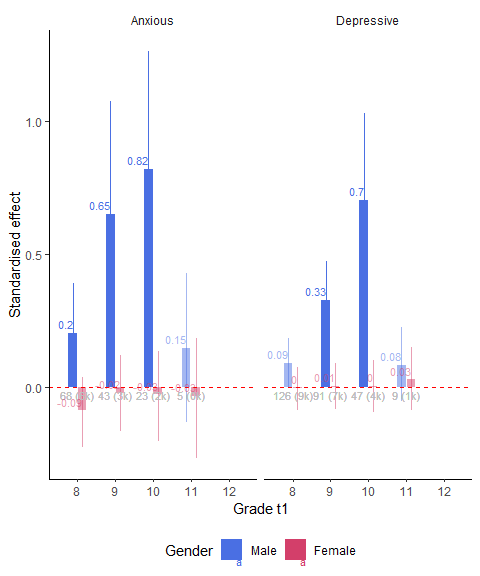  Figure S19: Effects of social media use on anxiety and depression symptoms stratified by sex and age group. The figure shows standardized effects, i.e. by how many standard deviations the outcome increases when the exposure increases by one standard deviation. Vertical lines indicate 95% Credible Intervals. Effect estimates where the Credible Interval includes 0 are shown in faint color. Numbers besides credible intervals are effect sizes. Numbers below or above bars in indicate number of county-cohorts (pupils) included in the analysis. |

Bürkner, Paul-Christian. 2017. “Brms: An r Package for Bayesian Multilevel Models Using Stan.” *Journal of Statistical Software* 80 (1): 1–28. <https://doi.org/10.18637/jss.v080.i01>.

Carpenter, Bob, Andrew Gelman, Matthew D. Hoffman, Daniel Lee, Ben Goodrich, Michael Betancourt, Marcus Brubaker, Jiqiang Guo, Peter Li, and Allen Riddell. 2017. “Stan: A Probabilistic Programming Language.” *Journal of Statistical Software* 76 (1): 1–32. <https://doi.org/10.18637/jss.v076.i01>.

Gelman, Andrew, and Jennifer Hill. 2006. *Data Analysis Using Regression and Multilevel/Hierarchical Models*. Cambridge University Press.

Mundlak, Yair. 1978. “On the Pooling of Time Series and Cross Section Data.” *Econometrica* 46 (1): 69–85. <http://www.jstor.org/stable/1913646>.

Neuhaus, J. M., and J. D. Kalbfleisch. 1998. “Between- and Within-Cluster Covariate Effects in the Analysis of Clustered Data.” *Biometrics* 54 (2): 638–45.

Robins, James M. 1986. “A New Approach to Causal Inference in Mortality Studies with a Sustained Exposure Period—Application to Control of the Healthy Worker Survivor Effect.” *Mathematical Modelling* 7 (9–12): 1393–1512.
